# Multi-Omics Mining of Characteristic Quality Factors Boosts the Brand Enhancement of the Geographical Indication Product—Pingliang Red Cattle

**DOI:** 10.3390/foods14101770

**Published:** 2025-05-16

**Authors:** Jing Liu, Yu Zhu, Xiaoxia Liu, Juan Zhang, Chuan Liu, Yan Zhao, Shuming Yang, Ailiang Chen, Jie Zhao

**Affiliations:** 1 State Key Laboratory for Quality and Safety of Agro-Products, Institute of Quality Standards and Testing Technology for Agro-Products, Chinese Academy of Agricultural Sciences, Beijing 100081, China; lj0125s@163.com (J.L.); lxxiagood@163.com (X.L.); 15110679191@yeah.net (J.Z.); 17390957656@163.com (C.L.); zhaoyan01@caas.cn (Y.Z.); yangshuming@caas.cn (S.Y.); 2 Gansu Food Inspection and Research Institute, Lanzhou 730030, China; sspjyyjyzy@163.com; 3 Institute of Quality Standard and Testing Technology, Beijing Academy of Agriculture and Forestry Sciences, Beijing 100080, China

**Keywords:** metabolome, transcriptome, meat quality, Pingliang Red Cattle

## Abstract

Pingliang Red Cattle, a renowned geographical indication product in China, is distinguished by its superior meat quality, yet the scientific basis for its unique attributes remains underexplored. This study integrated metabolomic and transcriptomic analyses to elucidate the biochemical and physiological factors underlying the enhanced flavor, color stability, and tenderness of Pingliang Red Cattle beef compared to Qinchuan and Simmental cattle. Metabolomic profiling revealed significantly elevated levels of inosine monophosphate (IMP, 2.86–3.96× higher) and glutathione (GSH, 2.42–5.43× higher) in Pingliang Red Cattle, contributing to intense umami flavor and prolonged meat color retention. Notably, ergothioneine (EGT), a potent antioxidant, was identified for the first time in Pingliang Red Cattle beef, with concentrations 2.55× and 4.25× higher than in Qinchuan and Simmental, respectively. Transcriptomic analysis highlighted the upregulation of 21 tenderness-related genes (e.g., *FABP3*, *PRDX6*, *CAST*) and key enzymes in purine and glutathione metabolism pathways (e.g., *PDE4D*, *ADSL*, *GGT1*), correlating with meat tenderness and the improved meat quality. Additionally, Pingliang Red Cattle’s natural forage-rich diet and low-density rearing practices were critical in enhancing these traits. These findings provide a scientific foundation for Pingliang Red Cattle’s premium quality, offering actionable insights for GI product branding, quality optimization, and market competitiveness. The multi-omics approach established here serves as a paradigm for quality assessment and improvement of other GI agricultural products, bridging traditional reputation with molecular evidence.

## 1. Introduction

Geographical indication products are often highly favored by consumers for their superior quality and unique characteristics. These products are typically closely associated with specific geographical regions, where the natural environment, traditional craftsmanship, and cultural heritage collectively contribute to their distinct attributes [1]. However, despite their high market potential, many of these products suffer from a lack of clear definition regarding their specific features, which limits their market promotion and further development. The absence of a solid scientific basis to support their uniqueness makes it difficult for these products to stand out in a competitive market [2]. Pingliang Red Cattle is a well-known geographical indication product among the general public [3]. Pingliang Red Cattle beef is renowned for its vibrant color, delicious taste, and tender texture. The marble score of Pingliang Red Cattle beef is greater than or equal to 4.5 and the taste is comparable to that of “Japanese Black Cattle” meat [4]. From a nutritional perspective, beef is an excellent source of high-quality protein, essential amino acids, bioavailable iron (heme iron), zinc, and B vitamins, which play crucial roles in muscle maintenance, immune function, and metabolic health [5]. According to a previous study, compared to other similar types of beef, the Pingliang Red Cattle beef has 18.92% higher total content of flavor precursor amino acids; it also contains 11.58% more oleic acid and 11.06% more linoleic acid. Additionally, the content of the aroma compound 1-octen-3-ol is 55.85% higher than other similar types of beef [6].

However, for a long time, the scientific foundation behind these desirable sensory qualities was unclear. This situation not only affects the further breeding and quality improvement of the product but also hinders brand enhancement and the realization of premium pricing. To overcome these challenges, it is necessary to conduct scientific research to uncover the specific reasons behind its superior qualities and to leverage these scientific findings to strengthen brand building and enhance market competitiveness. Such efforts can not only bring broader market prospects for Pingliang Red Cattle but also provide a successful model for other geographical indication products, helping them better showcase their unique value, and aiming to achieve the goal of premium quality at a fair price.

In recent years, the development of omics technologies has provided effective tools for uncovering characteristic factors in foods. Particularly, metabolomics and transcriptomics technology have emerged as robust tools in the agricultural and food research fields, offering comprehensive insights into the complex biological processes that underpin food safety, quality, and sustainability. Untargeted metabolomics, leveraging high-resolution mass spectrometry (HRMS) platforms, has been developing rapidly and boasts a broad range of applications, it could detect and quantify as many metabolites as possible within a biological sample, facilitating the discovery of novel metabolic pathways or biomarkers. In recent years, untargeted metabolomics analysis has been successfully applied in meat freshness evaluation [7], food safety assessment [8], food fraud identification [9], monitoring of component changes during food processing [10], and so on. Along with the continuous advancements in the Next-generation sequencing (NGS) technology, transcriptomics has profoundly deepened our comprehension of how genes are expressed within organisms, revealing intricate patterns and regulatory mechanisms that govern cellular functions [11]. At present, transcriptomics has been widely used in many fields, such as disease diagnosis and drug discovery [12], crop and animal breed improvement [13] and plant-pathogen interactions [14], etc. Additionally, the integration of metabolomics with transcriptomics, provide a systems-level understanding by analyzing their metabolites and gene expression profiles, which offers unprecedented opportunities for uncovering the distinctive qualities of Geographical Indication products. In recent years, livestock products [15], agricultural products [16], microbial fermentation, and pharmacological discovery [17] have greatly benefited from combined metabolomic and transcriptomic analyses.

In this study, metabolomic and transcriptomic analysis were integrated to uncover the distinctive quality indicators of Pingliang Red Cattle and the underlying gene expression mechanism. Results showed that Pingliang Red Cattle beef exhibit more intense umami flavor, longer-lasting color, and tenderer texture than the Qinchuan and Simmental cattle beef. Moreover, this study also made the first discovery of the presence of ergothioneine, a bioactive alkaloid and potent natural antioxidant, within Pingliang Red Cattle beef. Finally, this study analyzed the relationship between these distinctive quality traits and environmental feed factors, providing a paradigm for the quality exploration of Geographical Indication products.

## 2. Materials and Methods

### 2.1. Chemicals and Reagents

Both formic acid and methanol (chromatographically pure) were purchased from Beijing Dima Technology Co., Ltd. (Beijing, China). Acetonitrile (chromatographically pure) was obtained from Thermo Fisher Scientific (Fair Lawn, NJ, USA). Ultrapure water was obtained using a Milli-Q Integral Water Purification System (Millipore, Billerica, MA, USA). A TransZolUp Plus RNA Kit (Hangzhou Chuangshi Biotechnology Co., Ltd., Hangzhou, China) was used to extract total RNA. Reverse transcription was performed using an IST Strand cDNA Synthesis Kit (Baori Medical Biotechnology Co., Ltd., Beijing, China). SYBR FAST qPCR Kit Master Mix (2×) Universal (Annolun Biotechnology Co., Ltd., Beijing, China) was used for quantitative polymerase chain reaction (qPCR) validation.

### 2.2. Sample Collection and Preparation

Pingliang Red Cattle samples were obtained from four certified local feeding business from Pingliang city (China), where all cattle were free-range grazing without confinement in modern barns, Qinchuan and Simmental cattle samples (captive) were used as a control. The Simmental cattle were purchased from Horqin Cattle Industry Co., Ltd. (Inner Mongolia, China). To enhance the universality and reliability of this study, we selected cattle of the same breed from different pastures without imposing any deliberate restrictions on feeding methods, age, weight, or other variables. This approach could better reflect the variability and complexity under actual farming conditions, ensuring the broad applicability of the study results. Additionally, we concurrently collected and analyzed relevant information on the rearing of Pingliang Red Cattle, including feed types, feed consumption rates, and rearing methods. All samples (approximately 500 g each) were taken from the loin of healthy bulls that had not received antibiotic treatment for at least 3 months before sampling. Twenty samples were isolated from Pingliang Red Cattle and 15 samples were isolated from each group of control cattle (Qinchuan and Simmental). Information on samples collected from the three beef cattle breeds is shown in Table S1. From each of the three sample groups, four samples were randomly selected. These selected samples were then cut into 8–10 g pieces using scalpels and stored at –80 °C until they were subjected to transcriptome analysis. The remaining meat samples were minced using a meat grinder and stored at −20 °C until further analyses.

### 2.3. Metabolomic Profiling

Metabolites were extracted as previously described [18], with some modifications. Each tenderloin sample (weighing 150 mg, stored at −20 °C) was placed in a 2 mL centrifuge tube, and 1 mL of pre-frozen methanol/water solution (1/1, *v*/*v*) was added. The samples were ultrasonically treated in a water bath (20 °C) for 10 min, vortexed for 5 min, and centrifuged at 14,500× *g* for 20 min (4 °C). The supernatant was filtered through a 0.22 µm polytetrafluoroethylene membrane to obtain the final product. For each beef sample, quality control (QC) samples were prepared by equal pooling to monitor the stability and robustness of the data based on a previously described method [19]. A QC sample was inserted and measured after measuring every eight test beef samples during instrumental analysis. All samples were analyzed using a UPLC system equipped with a Q-TOF mass spectrometer (TripleTOF 6600, AB Sciex, Framingham, MA, USA). The solvent system used for UPLC comprised mobile phase A of 0.1% formic acid in water and mobile phase B of 0.1% formic acid in methanol. Chromatographic separation was performed using a C_18_ column (Zorbax Eclipse C_18_, 3.0 mm × 150 mm, 1.8 μm, Agilent, Foster City, CA, USA), which was maintained at 40 °C. Five µL of the extract was then injected at 4 °C, with the flow rate of the mobile phase being 0.3 mL/min. The elution gradient was as follows: 0.00–1.00 min, 90% A (10% B); 1.00–1.01 min, 90% A (10% B); 1.01–12.00 min,10% A (90% B); 12.00–12.10 min, 10% A (90% B); and 12.10–17 min, 90% A (10% B). TOF MS and product ion scanning were carried out simultaneously in the range of *m*/*z* 50–1000, using information-dependent acquisition (IDA) MS. The experimental parameters of TOF MS scanning in the positive-ion mode were set as follows: ion source, 5500 V; gas temperature, 500 °C; nebulizer gas (GS1), 50 psi; Tissue-Equivalent gas (GS2), 50 psi; declustering potential, 80 V; and collision energy (CE), 10 eV. The MS2 (50–1000 *m*/*z*) analysis was conducted at a CE of 35–15 eV, with the CE ramped up at an interval according to the CE spread value.

### 2.4. RNA Library Construction and RNA-Seq

Total RNA was extracted from samples in the three groups using a standard extraction method [20], and RNA integrity was accurately determined using a Bioanalyzer Agilent 2100 system (Agilent Technologies, CA, USA). The qualified RNA from each sample was used for library construction and RNA-seq by NuoheZhiyuan Biotechnology Co., Ltd. (Beijing, China). The cDNA library was sequenced on an IlluminaNovaSeq platform (Beijing, China). Four samples from each group were selected as biological replicates.

To obtain high-quality clean reads, the image data obtained from Illumina sequencing were converted into raw reads, and sequencing errors, low-quality reads, and sequence connectors in raw reads were removed. Q20 and Q30 scores and GC content were calculated to ensure the reliability of all subsequent clean data analyses. A reference genome index was created using HISAT2 v2.0.5 and then the paired clean reads were compared to the reference genome. Reads mapped to each gene were then studied, and the fragments per kilobase of transcript per million mapped reads of each gene value was calculated according to the length of the gene. Differentially expressed genes (DEGs) were identified using the DESeq2 method. cDNA obtained from RNA transcripts from beef tenderloin tissues in all three groups was used as a qPCR template, and the 18S rRNA gene was used as a reference gene to design primers. The expression levels of 10 genes were quantified using a QuantStudio6 Flex Real-Time PCR system. The genes analyzed and primers used are shown in Table 1, and the thermal cycling procedure is shown in Table 2. Each gene was analyzed four times by qPCR.

### 2.5. Statistical Analysis

MS-DIAL (version 4.90) was used to process the mass spectrum data, and the compounds lacking MS/MS (tandem mass spectrometry) data and those with response values below 5000 were excluded from the analysis (Table S2). Microsoft Office Excel 2021 was used to filter out the MS1 peaks with a detection rate below 80%, and relative standard deviation (RSD) greater than 30% of QC samples, and perform Student’s *t*-test analysis (after confirming the data met parametric assumptions including normal distribution and homogeneity of variance) and fold change analysis. The MS2 information of potential markers obtained by IDA was imported into MS-FINDER Version 3.52 software (Table S2), annotated using three major databases (HMDB, and LipidMaps), and analyzed to screen for significantly differential metabolites (SDM) of Pingliang Red Cattle. The screening criteria for SDM was fold change (FC) of ≥2 or ≤0.5, an adjusted *p*-value < 0.05, and a Variable Importance in Projection (VIP) value > 1. The biological functions and metabolic pathways of the SDM were analyzed using the Gene Ontology (GO) and Kyoto Encyclopedia of Genes and Genomes (KEGG) databases, respectively. Principal component analysis (PCA) and orthogonal partial least-squares discriminant analysis (OPLS-DA) were performed using the SIMCA^®^ of Umetrics company (Umea, Sweden). A heatmap and volcano plot were created using the MetaboAnalyst Software (https://www.metaboanalyst.ca/) (accessed on 15 October 2024) [21]. DESeq2 software (1.20.0) was used to perform the differential expression analysis of two comparison samples; genes with expression levels meeting the criteria *p* ≤ 0.05 and log2 fold change > 1 were designated as differentially expressed genes (DEG). The relative expression level of gene was calculated using the 2^−ΔΔCT^ method.

## 3. Results and Discussion

### 3.1. Identification of Differential Metabolites

Metabolomics based on mass spectrometry offers significant advantages due to its high selectivity and sensitivity, providing unparalleled convenience for identifying metabolites from samples of different origins. This provides insights into the distinctive flavors, nutritional profiles, and other quality attributes that characterize agricultural products originating from this region. Finally, 4876 original peaks were reserved. The total ion current (TIC) chromatograms of six QC samples exhibit high overlap, as shown in Figure S1, indicating good stability of the mass spectrometry signals. The high stability of the instrument ensures the repeatability and reliability of the data, providing a critical foundation for robust analytical results. Furthermore, as evident from the provided TIC chromatograms, the chromatographic peaks exhibit high resolution and moderate response intensity. This suggests that the metabolites extracted from the beef samples were well-separated, indicating effective extraction and analysis procedures.

**Figure 1:**
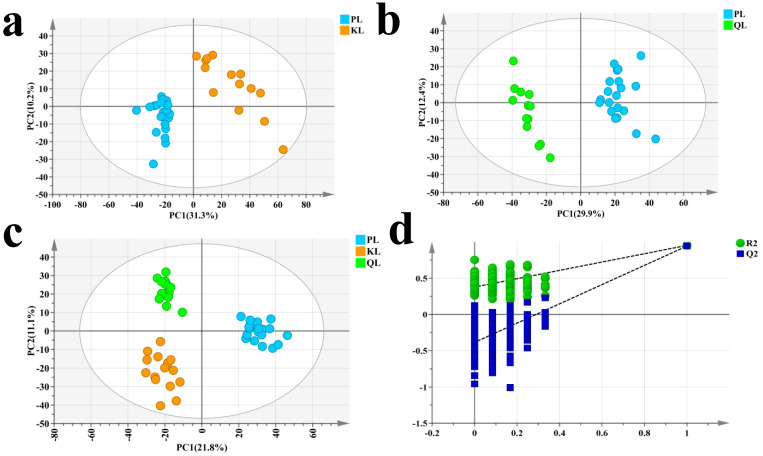
Multivariate analysis of beef metabolites across cattle breeds (PL, KL, QL). (**a**) PCA score plot of PL vs. KL metabolites. (**b**) PCA score plot of PL vs. QL metabolites. (**c**) OPLS-DA score plot showing metabolic discrimination. (**d**) Displacement test of metabolic variations. Abbreviations: PL, Pingliang Red Cattle; KL, Simmental; QL, Qinchuan; PCA, principal component analysis; OPLS-DA, orthogonal partial least squares discriminant analysis.

Principal Component Analysis (PCA) is an unsupervised pattern recognition method for multidimensional data statistical analysis. This method compresses the original data into a several principal components to describe the characteristics of the dataset. By performing PCA on the samples, we can gain preliminary insights into the overall metabolic differences between groups and the variability within each group [22]. The PCA results, illustrated in Figure 1a,b, demonstrate the separation trends of the metabolome between Pingliang Red Cattle and Simmental cattle, and Pingliang Red Cattle and Qinchuan cattle, suggesting that there are distinct differences in the metabolic profiles between Pingliang Red Cattle and the two other cattle.

Orthogonal partial least-squares discriminant analysis (OPLS-DA) combines Orthogonal Signal Correction (OSC) with Partial Least Squares Discriminant Analysis (PLS-DA). This approach allows for the filtering of variables by removing unrelated variations during modeling, thereby enhancing the selection of differential variables [23]. Therefore, an OPLS-DA model was constructed based on the metabolite data from the three sample groups, as shown in Figure 1c. The results further elucidates the distinct differences in metabolic profiles between the three groups, and also highlights that the Pingliang Red Cattle exhibit noticeable differences at the metabolite level compared to the Simmental and Qinchuan cattle. To validate the accuracy of the model, we conducted 200 permutation tests, as shown in Figure 1d, all the blue Q^2^ values and green R^2^ values on the left are lower than the original points on the right, indicating the validity of the original model. Furthermore, the R^2^Y value is 0.912 and the Q^2^ value is 0.796 in the model. The R^2^Y value approaches 1, the accuracy of the model increases, and Q^2^ value closer to 1 indicates greater predictive capacity in the original model [23]. So, the aforementioned results demonstrate that the model possesses excellent reliability and predictive capability.

**Figure 2:**
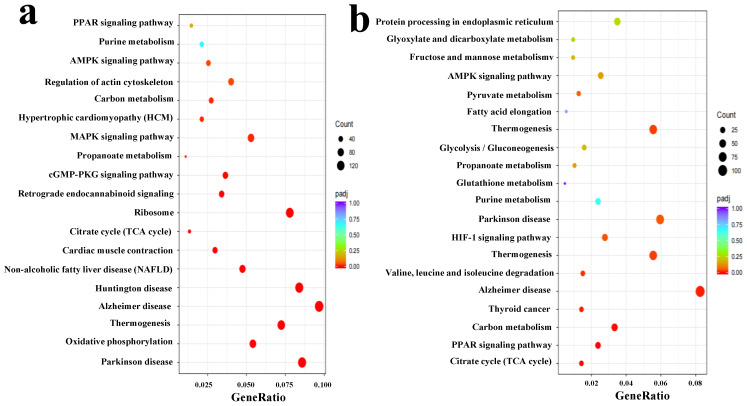
KEGG pathway enrichment analysis of significantly differential metabolites (SDM) in different beef cattle breeds. (**a**) KEGG pathway enrichment diagram of SDM in PL and KL. (**b**) KEGG pathway enrichment diagram of SDM in PL and QL. Abbreviations: KEGG, Kyoto Encyclopedia of Genes and Genomes; PL, Pingliang Red Cattle; KL, Simmental; QL, Qinchuan; SCM, significantly changed metabolites.

**Figure 3:**
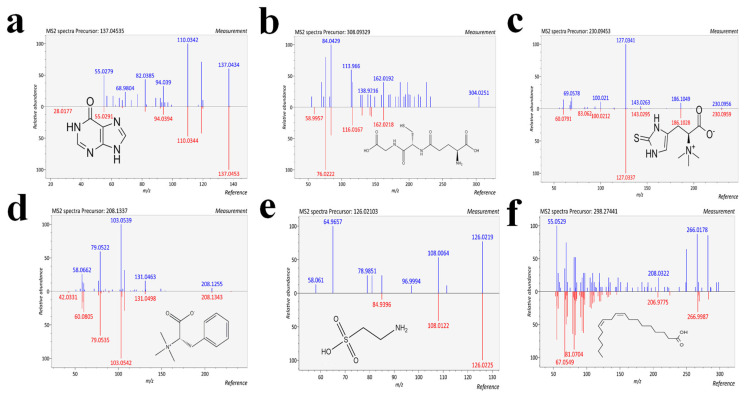
Annotation of unique biomarkers in Pingliang Red Cattle. Mirror plots and structures of (**a**) hypoxanthine, (**b**) glutathione, (**c**) ergothioneine, (**d**) taurine, (**e**) linoleic acid, and (**f**) phenylalanine betaine.

Based on the Student’s *t*-test and fold change (FC) analysis, and the OPLS-DA model, the Simmental and Qinchuan cattle were used as control samples to identify significant differential metabolites (SDM) with Pingliang Red Cattle. The screening criteria are as follows: FC of ≥2 or ≤0.5, an adjusted *p*-value < 0.05, and a Variable Importance in Projection (VIP) value > 1. By combining results of the MS-DIAL (a data-independent acquisition method) mirror image, 36 SDM were preliminarily screened as candidates distinguishing Pingliang Red Cattle and Simmental cattle (Table S3), and 28 SDM were preliminarily distinguished between Pingliang Red Cattle and Qinchuan cattle (Table S4). Among the SDM identified between Pingliang Red Cattle and Simmental cattle, 35 were significantly annotated to 32 pathways (*p* < 0.05; Figure 2a, Table S5); those identified SDM between Pingliang Red Cattle and Qinchuan cattle, 28 were significantly annotated to 18 pathways (*p* < 0.05; Figure 2b, Table S6). Of particular note, purine metabolism and glutathione metabolism are key metabolic pathways shared by Pingliang Red Cattle between Simmental and Qinchuan cattle, highlighting their significant research importance. Moreover, 30 SDM exhibited relatively higher levels in Pingliang Red Cattle samples, including 12 amino acids, 5 fatty acids, 5 alkaloids, 3 nucleotides, 2 purines, and 4 other metabolites (Table S7), such as hypoxanthine and its derivatives (IMP), glutathione (GSH), ergothioneine (EGT), taurine, linoleic acid and phenylalanine betaine. The mirror images and structural formulae of these metabolites are shown in Figure 3a–f, respectively. These metabolites play an important role in determining the flavor, taste, color, and antioxidant properties of meat.

### 3.2. Identification of DEG and Verification of Transcriptomic Data

**Figure 4:**
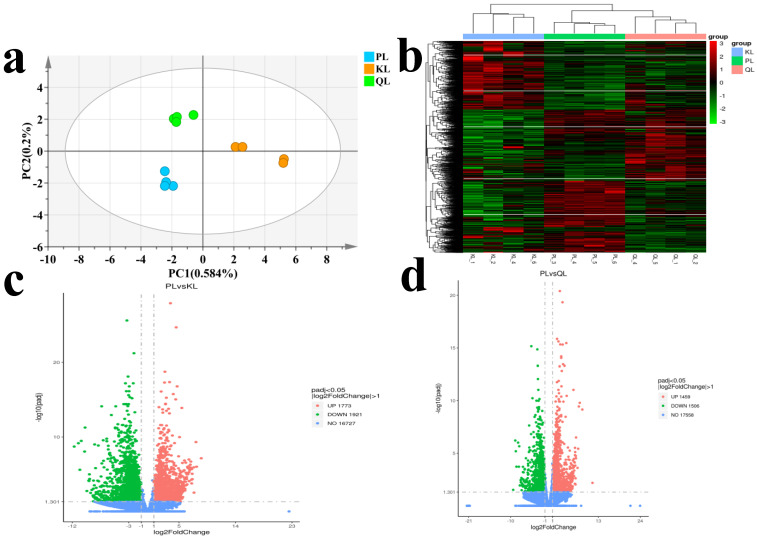
Identification and analysis of DEG among Pingliang Red Cattle, Simmental, and Qinchuan cattle. (**a**) PCA of identified genes. The red, green, and blue dots represent PL, KL and QL, respectively. (**b**) Heatmap of DEG. (**c**) Volcano plot of DEG (PL vs. KL). (**d**) Volcano plot of DEG (PL vs. QL). Red and dark green dots represent significantly upregulated and downregulated genes, respectively, and black dots represent genes with no significant changes. Abbreviations: PL, Pingliang Red Cattle; KL, Simmental; QL, Qinchuan.

Initially, we evaluated the quality of the RNA from the samples. The concentration and integrity assessment of sample RNA confirmed its suitable purity and integrity for subsequent sequencing and associated experiments (Table S8 and Figure S2). After removing sequencing adapters and low-quality reads, an average of 44,089,758 clean reads were obtained (Table S9). Sequence comparison showed that the 12 samples (four samples from each of the three cattle groups) had Q20 values (sites with base correctness > 99%) greater than 97% and Q30 values (sites with base correctness > 99% in the test data) greater than 94%, demonstrating the high quality of the sequencing data and their suitability for use in downstream analyses. To assess the reliability and representativeness of the samples, the squared Pearson correlation coefficient (R^2^) was analyzed, the results were as shown in Figure S3. Within-group samples with an R^2^ closer to 1 exhibit higher similarity in expression patterns, indicating more reliable results. Moreover, the within-group R^2^ being greater than the between-group R^2^ demonstrates that the selected samples are representative of their respective groups. Then, we performed PCA on the gene expression levels in each of the samples, as shown in Figure 4a. The PCA results demonstrate a clear and significant separation trend among the three groups of samples, highlighting the distinct gene expression profiles of Pingliang Red Cattle, Simmental Cattle, and Qinchuan Cattle. This separation suggests notable genetic expression differences between these breeds. Further clustering heatmap results show distinct groupings of gene expression patterns among Pingliang Red Cattle, Simmental Cattle, and Qinchuan Cattle, consistent with the PCA findings (Figure 4b).

**Figure 5:**
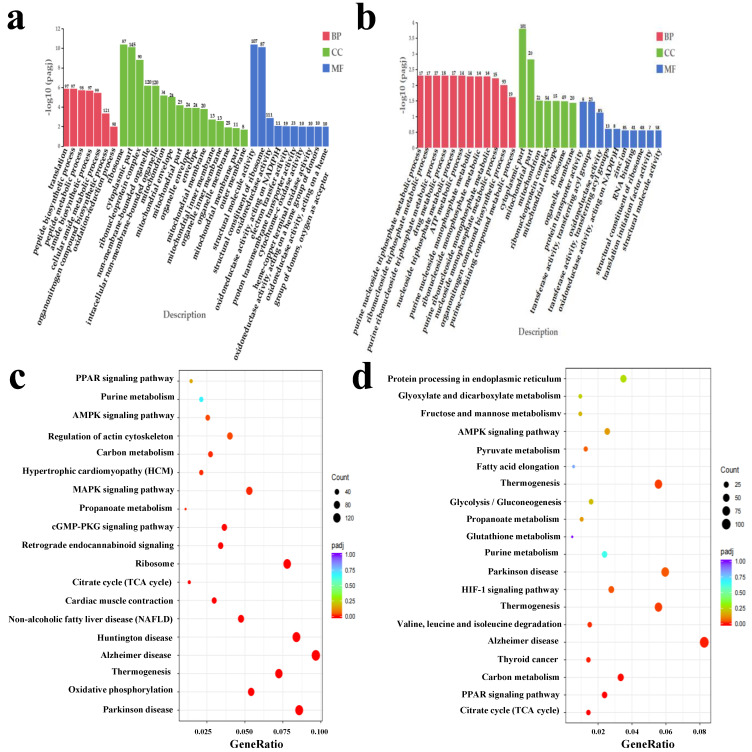
Functional enrichment analysis of differentially expressed genes comparing PL with KL and QL groups. (**a**) Significant GO terms for PL and KL. (**b**) Significant GO terms for PL and QL. (**c**) Significant KEGG pathways in PL and KL. (**d**) Significant KEGG pathways in PL and KL. Abbreviations: PL, Pingliang Red Cattle; KL, Simmental; QL, Qinchuan; BP, biological process; CC, cellular components; MF, molecular functions; GO, gene ontology; KEGG, Kyoto Encyclopedia of Genes and Genomes.

Differentially expressed genes (DEGs) were identified using the DESeq2 method, applying stringent filtering criteria: FC of ≥2 or ≤0.5 and an adjusted *p*-value < 0.05. These thresholds ensured that only genes with significant expression differences between the groups were selected for further analysis. In total, 3694 DEG were screened in the comparison between Pingliang Red Cattle and Simmental cattle, including 1773 upregulated genes and 1921 downregulated genes in Pingliang Red Cattle samples (Figure 4c). And 2695 DEG were identified in the comparison between Pingliang Red Cattle and Qinchuan cattle, including 1459 upregulated genes and 1506 downregulated genes in Pingliang Red Cattle samples (Figure 4d). To gain a deeper understanding of the biological roles and pathways associated with the DEGs, Gene Ontology (GO) enrichment analysis to categorize gene functions and Kyoto Encyclopedia of Genes and Genomes (KEGG) pathway analysis have been conducted to explore the involvement of these genes in specific metabolic and signaling pathways. These analyses provided valuable insights into the molecular mechanisms underlying the observed expression differences. The results were shown as Tables S10 and S11 and Figure 5a–d. The DEGs between the Pingliang Red Cattle and Simmental groups were enriched in 7 biological processes (BP), 16 cellular components (CC), and 10 molecular functions (MF). The DEGs between the Pingliang Red Cattle and Qinchuan groups were enriched in 12 BP, 7 CC, and 2 MF. Among these, many terms were related to beef quality, such as “peptide biosynthetic process” (GO: 0043043) and “purine nucleoside triphosphate metabolic process” (GO: 0009144). “Cellular components” and “ribosome types” were the main representatives of the CC terms, and “binding” and “enzyme activity” were dominant among the significantly enriched MFs. In addition, a series of KEGG signaling pathways related to beef quality were identified, including “purine metabolism” (bta00230), “glutathione metabolism” (bta00480), and “the PPAR signaling pathway” (bta03320). We therefore further focused on the GO terms and DEG involved in these KEGG pathways. The genes phosphodiesterase 4D (*PDE4D*), peroxiredoxin 6 (*PRDX6*), and 5-aminoimidazole-4-carboxamide ribonucleotideformyltransferase/IMP cyclohydrolase (*ATIC*) emerged as candidates most likely to have an important role in the taste and flavor of beef.

**Figure 6:**
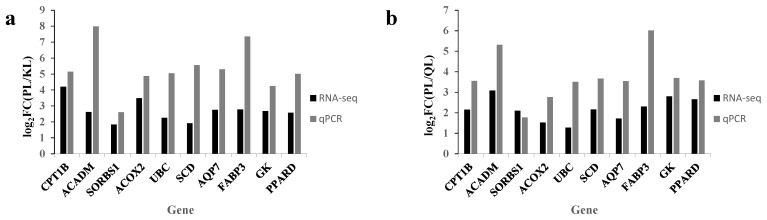
Real time quantitative PCR verification of randomly selected differentially expressed genes. (**a**) Pingliang Red Cattle and Simmental cattle. (**b**) Pingliang Red Cattle and Qinchuan cattle. Abbreviations: *CPT1B*, carnitinepalmitoyltransferase 1B; *ACADM*, acyl-CoA dehydrogenase medium chain; *SORBS1*, sorbin and SH3 domain containing 1; *ACOX2*, acyl-CoA oxidase 2; *UBC*, ubiquitin C; *SCD*, stearoyl-CoA desaturase; *AQP7*, aquaporin 7; *FABP3*, fatty acid binding protein 3; *GK*, glycerol kinase; *PPARD*, peroxisome proliferator activated receptor delta.

To verify the reliability of the RNA-seq data, 10 DEG (*CPT1B*, *ACADM*, *SORBS1*, *ACOX2*, *UBC*, *SCD*, *AQP7*, *GK*, *PPARD*, and *FABP3*) identified between the cattle groups were randomly selected for qPCR. As shown in Figure 6 and Table S12, the expression levels of the selected genes showed some deviations, but the overall trends were consistent, indicating that the RNA-seq analysis results were accurate and reliable.

### 3.3. Unveil Pingliang Red Cattle’s Distinctive Quality Traits

To comprehensively elucidate the unique quality attributes of Pingliang Red Cattle, an integrative analysis combining metabolomics, transcriptomics, specific feed composition, and rearing environment was conducted. Specifically, we identified two key metabolites that are closely associated with the umami flavor and color of beef: inosine monophosphate (IMP) and glutathione (GSH), as well as one high-content bioactive substance, ergothioneine (EGT), in Pingliang Red Cattle beef. Furthermore, transcriptomics analysis not only revealed the genetic basis behind the higher content of the aforementioned three metabolites but also identified a set of genes associated with meat tenderness, which was found to be significantly upregulated in Pingliang Red Cattle compared to both Qinchuan and Simmental cattle. Through this integrative analysis, we comprehensively elucidated the causes behind the superior quality of Pingliang Red Cattle, offering solid scientific data to support the explanation of their distinctive traits.

#### 3.3.1. IMP and Meat Flavor

**Figure 7:**
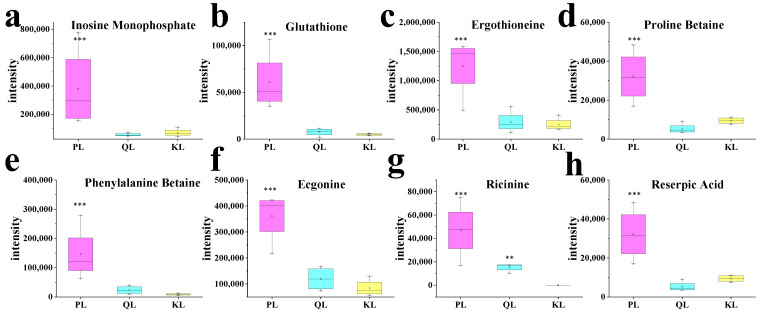
Contents of metabolites as potential markers for beef quality determined by liquid chromatography-mass spectrometry in Pingliang Red Cattle. (**a**) Inosine Monophosphate, (**b**) Glutathione, (**c**) Ergothioneine, (**d**) Proline Betaine, (**e**) Phenylalanine Betaine, (**f**) Ecgonine, (**g**) Ricinine, and (**h**) Linoleic acid. Asterisks indicate statistically significant differences: * *p* < 0.05, ** *p* < 0.01, *** *p* < 0.001.

IMP (inosinic acid) is a key umami compound that contributes significantly to the flavor characteristics of meat [24,25]. As the primary nucleotide responsible for meat’s savory taste, IMP and its derivatives play an essential role in determining overall flavor quality. Our metabolomics analysis demonstrated that the relative IMP content in Pingliang Red Cattle samples was 2.86 and 3.96 times higher than in Qinchuan and Simmental cattle samples, respectively (Figure 7a). These findings suggest that Pingliang Red Cattle possess superior flavor potential compared to the other breeds, which may translate to enhanced taste attributes. The elevated IMP levels could explain the more pronounced umami characteristics observed in this cattle breed.

**Figure 8:**
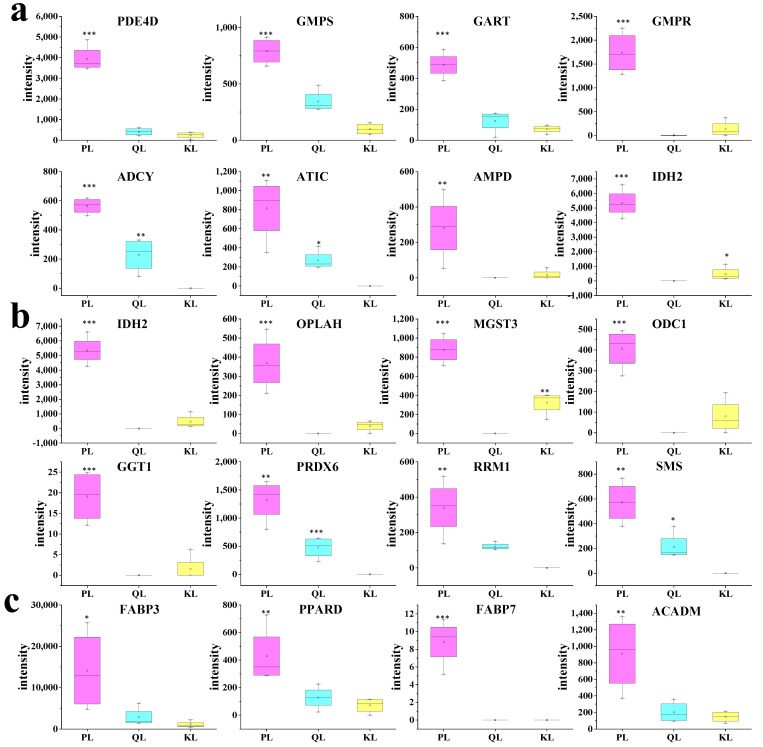
Comparison of gene transcript levels in Pingliang Red Cattle, Simmental, and Qinchuan cattle samples. (**a**) Purine metabolism-related genes. (**b**) Glutathione metabolism-related genes. (**c**) Shear force-related genes. Abbreviations: *PDE4D*, phosphodiesterase 4D; *GMPS*, guanine monophosphate synthase; *GART*, glycinamideribonucleotidetransformylase; *GMPR*, guanosine monophosphate reductase; *ADCY*, adenylatecyclase; *ATIC*, 5-aminoimidazole-4-carboxamide ribonucleotideformyltransferase/IMP cyclohydrolase; *AMPD*, adenosine monophosphate deaminase; *ADSL*, adenylosuccinatelyase; *IDH2*, isocitrate dehydrogenase 2; *OPLAH*, 5-oxoprolinase ATP-hydrolyzing; *MGST3*, microsomal glutathione S-transferase 3; *ODC1*, ornithine decarboxylase 1; *GGT1*, gamma-glutamyltransferase 1; *PRDX6*, peroxiredoxin 6; *RRM1*, ribonucleotidereductase catalytic subunit M1; *SMS*, spermine; *FABP3*, fatty acid binding protein 3; *PPARD*, peroxisome proliferator activated receptor delta; *FABP7*, fatty acid binding protein 7; *ACADM*, acyl-CoA dehydrogenase medium chain. Asterisks indicate statistically significant differences: * *p* < 0.05, ** *p* < 0.01, *** *p* < 0.001.

Through transcriptomics analysis of the purine metabolism pathway in cattle, compared to Simmental and Qinchuan cattle, Pingliang Red Cattle exhibited significantly increased transcription levels of 17 and 14 key genes, respectively, within the purine metabolism pathway. Among these, eight genes were commonly upregulated in both comparisons, including *PDE4D*, *GMPS*, *GART*, *GMPR*, *ADCY*, *ATIC*, *AMPD*, and *ADSL*. The relative expression levels of the eight genes, as depicted in Figure 8a.

For example of Phosphodiesterase 4D (*PDE4D*), the *PDE4D* has the Enzyme Commission (EC) number 3.1.4.53. The expression level of the *PDE4D* gene in Pingliang Red Cattle was 9.66 times higher than that in Qinchuan cattle and 16.04 times higher than in Simmental cattle. This substantial upregulation suggests enhanced cAMP-specific phosphodiesterase activity, which plays a crucial role in regulating intracellular signaling pathways related to muscle development and metabolism.

The formation of umami substances is regulated by several enzymes, including AMP deaminase (*AMPD*), adenylosuccinatelyase (*ADSL*), and *ATIC. AMPD1* catalyzes adenosine monophosphate hydrolysis and deamination to produce IMP, whereas ADSL catalyzes the initial synthesis and circulation of purine nucleotides and is the rate-limiting enzyme for IMP formation. ATIC is a biofunctional enzyme with aminoimidazole carbamoyl transferase and catalytic activities that regulates IMP synthesis [26]. Therefore, we have elucidated the intrinsic reasons for the superior flavor of Pingliang Red Cattle beef from both transcriptional and metabolic perspectives.

#### 3.3.2. GSH and Meat Color

Glutathione (GSH) is a tripeptide with γ-amide bonds and a sulfhydryl, composed of glutamic acid, cysteine, and glycine. GSH is a crucial antioxidant that plays a vital role in protecting cells from oxidative stress and damage. Beyond its antioxidant role, GSH is involved in several metabolic processes: such as (1) detoxification, conjugates with toxins and carcinogens, facilitating their excretion from the body [27]. (2) DNA synthesis and repair, contributing to genomic stability. (3) Immune function, facilitate the proliferation of lymphocytes and the production of cytokines [28]. Research has found that beef treated with a combination of GSH and a mixed gas of CO and O_3_ exhibited significantly better results in terms of total bacterial count, total volatile basic nitrogen (TVBN), and redness value (a*) during refrigerated storage compared to the group treated only with the mixed gas of CO and O_3_ [29]. The study results indicate that the presence of antioxidants can effectively maintain the bright color and remove off-flavors.

The metabolomics analysis results showed that the relative content of glutathione (GSH) in Pingliang Red Cattle samples is 5.43 times and 2.42 times higher than that in Qinchuan cattle and Simmental cattle, respectively, and the differences are statistically significant (Figure 7b).

Meanwhile, the transcriptomic analysis of the glutathione metabolism pathway in beef samples revealed that, compared to Qinchuan cattle and Simmental cattle, Pingliang Red Cattle samples had eight genes with extremely significant upregulation in their relative expression levels, and these expression levels were positively correlated with the glutathione content in the beef (Figure 8b). These genes are specifically identified as: *IDH2*, *OPLAH*, *MGST3*, *ODC1*, *GGT1*, *PRDX6*, *RRM1*, and *SMS*. The relative expression levels of these genes were more than two-fold higher in Pingliang Red Cattle compared to both Qinchuan cattle and Simmental cattle. Waerp et al. reported that upregulation of *MGST3* expression, which has GSH transferase activity and can catalyze various GSH-dependent reactions during GSH synthesis, is closely related to meat color. Glutathione may improve muscle water retention, meat color, and meat quality by reducing the damage of free radical reaction to cell structure and the oxidation degree of myoglobin [30]. So to improve glutathione content in meat, targeted intervention measures can be taken. Studies have shown that the glutathione level in slaughtered muscles is increased by supplementing the diet with hydroxymethionine 14 days before slaughter, and the oxidation of meat after slaughter is alleviated [31].

#### 3.3.3. DEGs and Meat Tenderness

Meat tenderness is a key indicator of beef quality and determines consumer satisfaction with the meat. Beef tenderness is categorized based on shear stress values as follows: tender and liked (<4.0 kg), acceptable (4.1–5.2 kg), and tough and disliked (>5.3 kg). The shear stress of the outer ridge of Pingliang Red Cattle beef is 2.72 kg, which is 19.58% lower than that of A3-grade snowflake beef purchased in the market [32]. The tenderness of beef is influenced by multiple factors, with cattle genotype playing a significant role alongside various environmental and management conditions. Research indicates that genetic factors contribute substantially to meat tenderness, though this characteristic is also notably affected by post-slaughter handling, feeding regimes, and processing techniques [33]. Genetic factors that determine beef tenderness mainly include specific variants of myogenic determination (*MYOD*), calmodulin kinase (*CaMK*), calpain (*CAPN*), calpain inhibitor (*CAST*), fatty acid binding protein (*FABP*), and *PRDX6*. In the study, transcriptomic analysis results show that, compared to Qinchuan and Simmental cattle, Pingliang Red Cattle have 21 significantly upregulated DEGs related to beef tenderness. These DEGs are as follows: *FABP3*, *PPARD*, *FABP7*, *ACADM*, *PRDX6*, *ACSL1*, *PLIN2*, *CPT1B*, *SORBS1*, *PDPK1*, *ACOX2*, *ACSL3*, *UBC*, *SCD*, *CPT2*, *AQP7*, *CD36*, *ACADL*, *GK*, *ME1*, *CAST.* Additionally, the relative expression levels of *FABP3*, *PPARD*, *FABP7*, and *ACADM* in Pingliang Red Cattle were significantly higher compared to Qinchuan and Simmental cattle (Figure 8c). Notably, the expression level of *FABP3* in Pingliang Red Cattle was 12.81-fold times higher than that in Simmental cattle. *CAST* activity is highly correlated with meat tenderness, and its average contribution to muscle shear force reaches up to 28.9%; thus, it has been proposed as a candidate gene for studying meat tenderness traits [34]. FABP proteins belong to a family of homologous small-molecule cytoplasmic proteins. Nine FABP members have been identified to date, among which heart- and adipocyte-specific members are considered candidate genes for studying intramuscular fat. *FABP3* polymorphisms significantly correlate with water content, tenderness, muscle scores, and intramuscular fat content [35]. PRDX6, an important antioxidant defense enzyme in animals, can regulate beef tenderness during postmortem ripening by regulating the level of oxidative stress in cells, thus serving as potential a biomarker of beef tenderness [36]). Notably, due to the varying expression levels of PRDX6 among cattle breeds, it can serve as a biomarker for beef tenderness. Specifically, PRDX6 exhibits a significant positive correlation with another well-known protein biomarker for beef tenderness, u-calpain (CAPN1). This suggests that *PRDX6* plays an important role in determining meat quality and could be used as an indicator for assessing beef tenderness across different cattle breeds [34].

### 3.4. Bioactive Compounds

Ergothioneine (EGT) is a rare amino acid that was first discovered in 1909, which was initially identified in ergot fungi (*Claviceps purpurea*), and EGT has since been found in various organisms, including bacteria, plants, and animals. EGT has many physiological functions, such as scavenging free radicals, detoxification, maintaining DNA biosynthesis, supporting normal cell growth, and enhancing cellular immunity [37]. Both the European Food Safety Agency and US Food and Drug Administration have affirmed the importance of EGT in functional foods and support that it can be safely added to pregnant women’s diet and children’s food [38]. Metabolomic analysis results show that the EGT relative content in Pingliang Red Cattle samples is significantly higher compared to Qinchuan and Simmental. Specifically, the EGT relatively content in Pingliang Red Cattle is 2.55 times higher than that in Qinchuan cattle and 4.25 times higher than that in Simmental cattle (Figure 7c). Subsequently, a confirmatory method for L-ergothioneine in beef samples was established using UPLC-Q-TOF-MS. The method was tested on solutions of L-ergothioneine standard and three types of beef samples. The results indicated that under positive ion scanning mode, compounds with primary ions (*m*/*z* 230.0954) and secondary ions were detected from beef samples of Red Pingliang cattle, Simmental cattle, and Qinchuan cattle. These findings matched the retention times, primary ions, and secondary ions of the L-ergothioneine standard solution, as shown in Figure 3c. Following this, according to the chromatographic and mass spectrometric conditions described in Section 2.3, quantitative analysis of L-ergothioneine in three types of beef samples was conducted using a standard solution of L-ergothioneine. A corresponding calibration curve was established: y = 347.08x + 302.09 (R^2^ = 0.9926). This curve demonstrated a good linear relationship for L-ergothioneine within the concentration range of 1.0 to 200.0 μg/L (Figure S4). To calculate the concentration of L-ergothioneine using a standard curve, the average concentration of L-ergothioneine in Pingliang Red Cattle was 16.6 μg/kg, in Simmental cattle it was 3.93 μg/kg, and in Qinchuan cattle it was 6.53 μg/kg. Based on this results, there were significant differences in the L-ergothioneine content among the three cattle breeds.

Although no experimental studies have shown that animals can synthesize ergothioneine (EGT), it is believed that they may absorb and retain it in their cells. Furthermore, transcriptomic analysis of samples from three cattle breeds did not identify any genes related to the synthesis or metabolism of EGT, nor their expression. This suggests that animals can only digest and absorb EGT, indicating that the EGT found in beef originates from the feed consumed by the animals. To determine the source of this component, we further investigated the feed sources of Pingliang Red Cattle. In contrast to the intensive feeding practices for Simmental and Qinchuan cattle, the feed composition for Pingliang Red Cattle typically consists of coarse feed (70%) and fine feed (30%). The coarse feed is derived from a wide variety of sources, including multiple plant stems, leaves, and natural pasture grasses, which serve as the primary contributors of ergothioneine.

The alkaloids detected in the meat are completely or partially derived from the digestion, absorption, and retention of these feeds by the animals. To understand the alkaloid content in the natural forage consumed by the cattle, our research team conducted a comprehensive analysis over two consecutive years. A total of 70 samples of natural forage were analyzed for their alkaloid content. The results indicated an overall detection rate of 42.86% for alkaloids, with 11 out of the 15 types of alkaloids being detectable. The total alkaloid content in the samples ranged from 10.40 to 106.99 g/kg. Further research has revealed that, in addition to EGT mentioned earlier, the bioactive alkaloids retained in Pingliang Red Cattle meat samples include: D-proline betaine, phenylalanine betaine, anabasine, ricinine, butyryl carnitine, and hematoporphyrin.

Our investigation also revealed that Pingliang Red Cattle are raised at low stocking densities with relatively low levels of intensification. This management practice contributes to higher animal welfare standards, as evidenced by the animals’ behavior. The improved welfare conditions have a positive influence on beef quality to some extent.

## 4. Conclusions

This study identified significant biochemical differences in Pingliang Red Cattle compared to Simmental and Qinchuan cattle, including: (1) elevated levels of flavor-related compounds (IMP and GSH), (2) upregulation of tenderness-associated genes, and (3) higher content of the bioactive compound ergothioneine (EGT). While these biochemical markers suggest potential implications for meat quality attributes, we acknowledge that actual sensory characteristics would require verification through dedicated evaluation methods. Through integrated metabolomics and transcriptomics analysis combined with feeding condition assessment, we have systematically characterized the distinctive biochemical profile of Pingliang Red Cattle. These findings provide technical references for understanding the molecular basis of geographical indication products and establish a foundation for future research incorporating sensory analysis to fully evaluate meat quality attributes.

## Figures and Tables

**Table 1:** Primer sequences used for real-time quantitative polymerase chain reaction.

Gene Name (Symbol)	Primer Sequences (5′–3′)	Product Length (bp)
Carnitine palmitoyltransferase 1B (*CPT1B*)	CCCAGGGAAGGACACAGATGT	149
	GGATCCTCTGGAACTGCATCTC	
Acyl-CoA dehydrogenase medium chain (*ACADM*)	GCTTGGGAAGTTGATTCTGGTC	224
	TGTTCACGGGCTATAATAAGCC	
Peroxisome proliferator-activated receptor delta (*PPARD*)	TGCAAAATCCAGAAGAAGAACC	171
	CTGGGGGTTGTGCTGACTC	
Acyl-CoA oxidase 2 (*ACOX2*)	TTCCTGTCTGGTGCCCAAATA	160
	GACGTTCATAGGCATGTCCATC	
Ubiquitin C (*UBC*)	CCGGACCGGGAGTTCAGT	172
	GGGATGCCTTCTTTTTCTTGTAT	
Stearoyl-CoA desaturase (*SCD*)	CCAGGGCACCCATCAGATAG	162
	TCCAAGGTGGTCTCGACA	
Sorbin and SH3 domain containing 1 (*SORBS1*)	TGTCCTGGAAGGAGGAGACATC	110
	CAGCTGGTATAAAATGCCTTGG	
Aquaporin 7 (*AQP7*)	TCCAAGGTGGTCTCGACA	204
	ACCTATGGTGACTCCGAAGC	
Fatty acid binding protein 3 (*FABP3*)	GCGTTCTCTGTCGTCTTTCC	169
	GATGATTGTGGTAGGCTTGGT	
Glycerol kinase (*GK*)	TAAGGAAATTCTGCAGTCTGTCT	139
	TAACTTGTCCCAGACTACAGTGG	
18S rRNA	CGGAACTGAGGCCATGATT	145
	CCTCCGACTTTCGTTCTTGAT	

**Table 2:** Program for real-time quantitative PCR polymerase chain reaction.

Procedure	Temperature (°C)	Time
Pre-incubation	95	5 min
Amplification (40 cycles)	95	3 s
	63	20 s
Melting curves	95	15 s
	60	15 s
	95	15 s

## Data Availability

The original data of this study are included in the article/Supplementary Material. Transcriptomic sequencing data are available through the NCBI Sequence ReadArchive (Bio Project ID: PRJNA1100214).

## Supplementary Materials

The following supporting information can be downloaded at: https://www.mdpi.com/article/10.3390/foods14101770/s1, Figure S1: The total ion current (TIC) chromatograms of six QC samples; Figure S2: Agilent 5400 test results for the integrity of sample RNA. (a) Pingliang Red cattle; (b) Simmental cattle; (c) Qinchuan cattle; Figure S3: The squared Pearson correlation coefficient (R²) within the three group samples; Figure S4: The calibration curve of L-ergothioneine; Table S1: The information of beef samples; Table S2: Summary of Key Processing Parameters; Table S3: Significantly distinct metabolites of PL&KL were discovered by LC-MS; Table S4: Significantly distinct metabolites of PL&QL were discovered by LC-MS; Table S5: KEGG pathway analysis in PL&KL DEGs; Table S6: KEGG pathway analysis in PL&KL DEGs; Table S7: High content of differential metabolites in Pingliang Red Bull; Table S8: Summary of RNA detection results in each group; Table S9: Summary table of quality assessment of RNA-seq sequencing data ofsamples; Table S10: The significant GO terms and KEGG pathways for differentially expressed genes (PLvsKL); Table S11: The significant GO terms and KEGG pathways for differentially expressed genes (PLvsQL); Table S12: Validation of randomly selected differentially expressed genes by qRT-PCR; Table S13: Comparison of gene transcript levels in Pingliang Red Bull, Simmental, and Qinchuan cattle samples.

## Abbreviations

The following abbreviations are used in this manuscript:

Acyl-CoA synthetase long-chain family member 1 (ACSL1), perilipin 2 (PLIN2), 3-phosphoinositide dependent protein kinase 1 (PDPK1), acyl-CoA synthetase long-chain family member 3 (ACSL3), carnitine palmitoyltransferase 2 (CPT2), Acyl-CoA dehydrogenase medium chain (ACADM); Adenylatecyclase (ADCY); Adenylosuccinatelyase (ADSL); Adenosine monophosphate deaminase(AMPD); 5-aminoimidazole-4-carboxamide ribonucleotideformyltransferase/IMP cyclohydrolase (ATIC); fatty acid binding protein 3 (FABP3); Fatty acid binding protein 7 (FABP7); Glycinamideribonucleotidetransformylase (GART); Gamma-glutamyltransferase 1 (GGT1); Guanosine monophosphate reductase (GMPR); Guanine monophosphate synthase (GMPS); Isocitrate dehydrogenase 2 (IDH2); Microsomal glutathione S-transferase 3 (MGST3); Ornithine decarboxylase 1 (ODC1); 5-oxoprolinase ATP-hydrolyzing (OPLAH); Phosphodiesterase 4D (PDE4D); Peroxisome proliferator activated receptor delta (PPARD); Peroxiredoxin 6 (PRDX6); Ribonucleotidereductase catalytic subunit M1 (RRM1); Spermine (SMS); Acyl-CoA dehydrogenase medium chain (ACADM); Acyl-CoA oxidase 2 (ACOX2); Aquaporin 7 (AQP7); Carnitinepalmitoyltransferase 1B (CPT1B); Glycerol kinase (GK); Peroxisome proliferator activated receptor delta (PPARD); Stearoyl-CoA desaturase (SCD); Sorbin and SH3 domain containing 1 (SORBS1); Ubiquitin C (UBC).
