# Supplementary material for: Multi-Omics Mining of Characteristic Quality Factors Boosts the Brand Enhancement of the Geographical Indication Product—Pingliang Red Cattle
Source: Foods. 2025 May 16;14(10):1770. doi: 10.3390/foods14101770 (PMC12111161; doi:10.3390/foods14101770)
Supplement: Supplementary file 1 [file foods-14-01770-s001.zip › foods-3561227-supplementary.pdf]

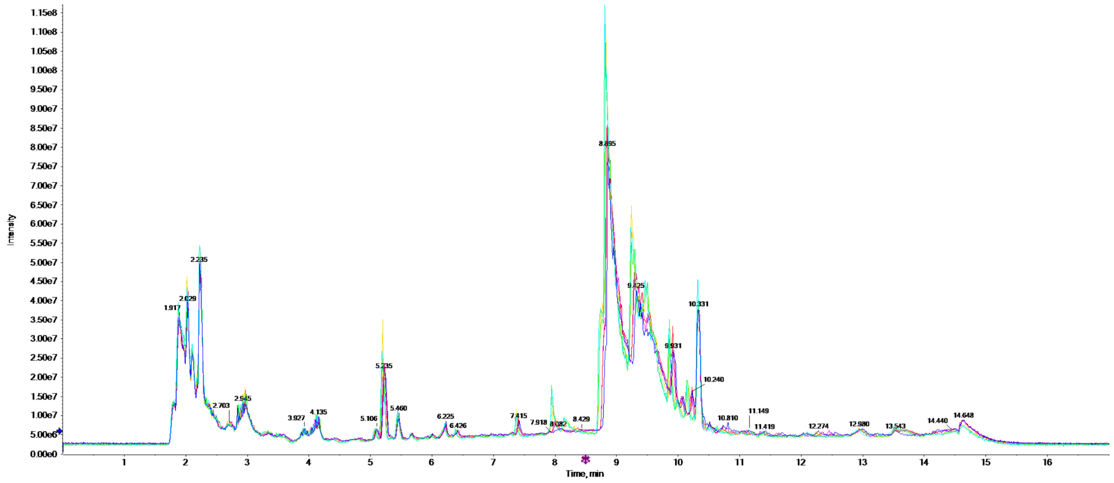

Figure S1. The total ion current (TIC) chromatograms of six QC samples

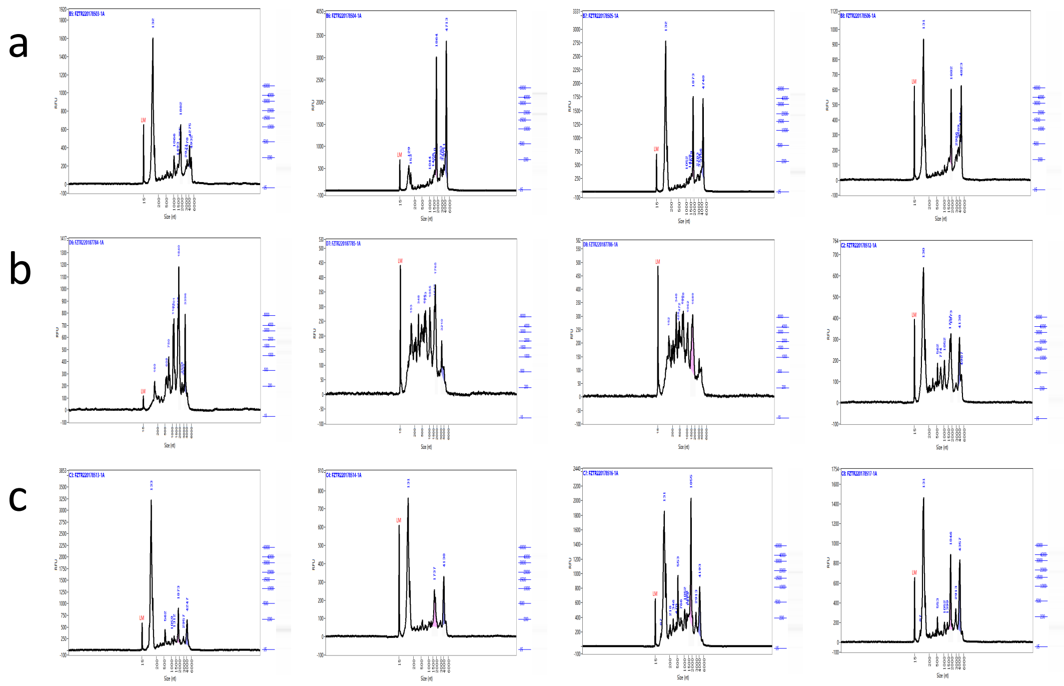

Figure S2. Agilent 5400 test results for the integrity of sample RNA. (a) Pingliang Red cattle; (b) Simmental cattle; (c) Qinchuan cattle.

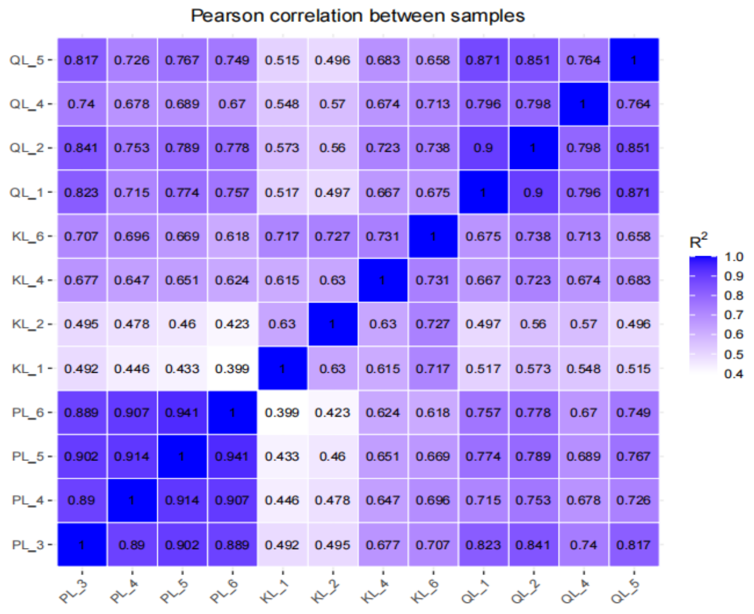

Figure S3. The squared Pearson correlation coefficient (R^2) within the three group samples. PL-Pingliang Red cattle; KL-Simmental cattle; QL-Qinchuan cattle.

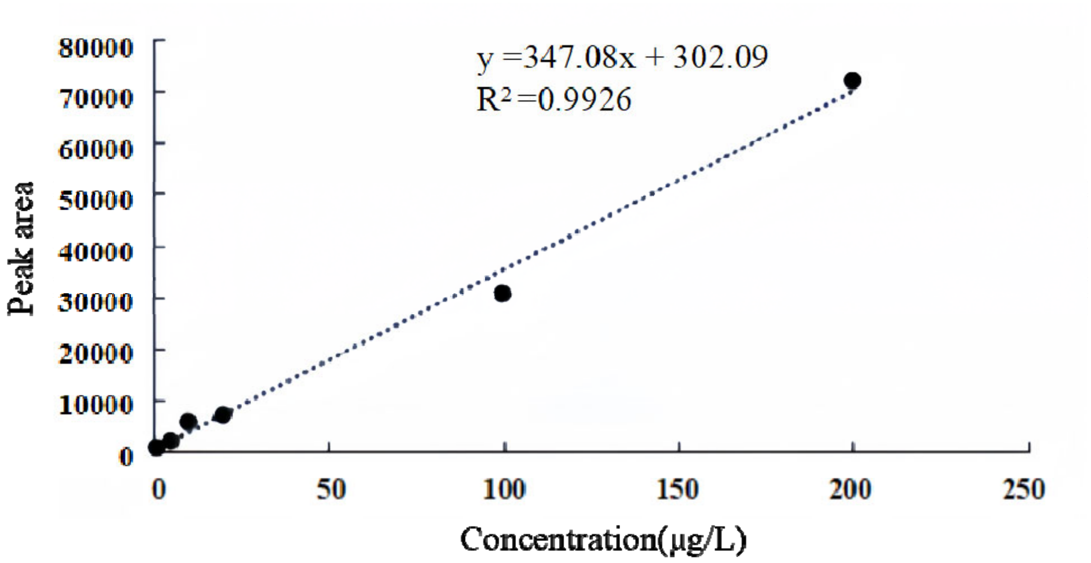

Figure S4. The calibration curve of L-ergothioneine
